# miRNA155-5P participated in DDX3X targeted regulation of pyroptosis to attenuate renal ischemia/reperfusion injury

**DOI:** 10.18632/aging.204692

**Published:** 2023-05-04

**Authors:** Yan Zhang, Xinghua Lv, Qian Fan, Feng Chen, Zhanhai Wan, Janvier Nibaruta, Hao Wang, Xiaoxia Wang, Yuan Yuan, Wenwen Guo, Yufang Leng

**Affiliations:** 1 Department of Anesthesiology, First Hospital of Lanzhou University, Lanzhou, Gansu, China; 2 The First Clinical Medical College of Lanzhou University, Lanzhou, Gansu Province, China; 3 Tianjin Eye Hospital and Eye Institute, Tianjin Key Lab of Ophthalmology and Visual Science, Nankai University Affiliated Eye Hospital, Nankai Eye Institute, Nankai University, Clinical College of Ophthalmology, Tianjin Medical University, Tianjin, China

**Keywords:** miR-155-5p, pyroptosis, NLRP3, DDX3X, caspase-1

## Abstract

Background: Renal ischemia/reperfusion injury (IRI) induced pathological damage to renal microvessels and tubular epithelial cells through multiple factors. However, studies investigated whether miRNA155-5P targeted DDX3X to attenuate pyroptosis were scarce.

Results: The expression of pyroptosis-related proteins (caspase-1, interleukin-1β (IL-1β), NOD-like receptor family pyrin domain containing 3 (NLRP3), and IL-18) were up-regulated in the IRI group. Additionally, miR-155-5p was higher in the IRI group comparing with the sham group. The DDX3X was inhibited by the miR-155-5p mimic more than in the other groups. DEAD-box Helicase 3 X-Linked (DDX3X), NLRP3, caspase-1, IL-1β, IL-18, LDH, and pyroptosis rates were higher in all H/R groups than in the control group. These indicators were higher in the miR-155-5p mimic group than in the H/R and the miR-155-5p mimic negative control (NC) group.

Conclusions: Current findings suggested that miR-155-5p decreased the inflammation involved in pyroptosis by downregulating the DDX3X/NLRP3/caspase-1 pathway.

Methods: Using the models of IRI in mouse and the hypoxia-reoxygenation (H/R)-induced injury in human renal proximal tubular epithelial cells (HK-2 cells), we analyzed the changes in renal pathology and the expression of factors correlated with pyroptosis and DDX3X. Real-time reverse transcription polymerase chain reaction (RT-PCR) detected miRNAs and enzyme-linked immunosorbent assay (ELISA) was used to detect lactic dehydrogenase activity. The StarBase and luciferase assays examined the specific interplay of DDX3X and miRNA155-5P. In the IRI group, severe renal tissue damage, swelling, and inflammation were examined.

## INTRODUCTION

Ischemia/reperfusion injury (IRI) was constructed by the restriction of blood flow and oxygen supply to kidneys. Unavoidable renal injury mostly occurred during organ transplantations, cardiac surgeries, and sepsis. All of these injuries exacerbate tissue damage by initiating inflammatory cascade responses that include the recruitment and activation of cytokines, chemokines, and immune cells [1–4]. IRI leads to acute kidney injury (AKI) (within 1-7 days) and sustained (>24 h) decline in renal function and an increase in mortality risk [5]. Renal IRI induced pathological damage to renal microvessels and tubular epithelial cells through multiple factors [6].

Pyroptosis is a galdermin-mediated programmed cell death [7]. Cells release large amounts of lactate dehydrogenase (LDH) during pyroptosis, which is often used as an indicator. Recent studies reported that pyroptosis associated with renal ischemia/reperfusion (I/R) is a compelling cause of renal tissue damage. Pyroptosis is induced by inflammasomes, which were innate immune sensors that recognize a wide range of exogenous (microbial molecules) and endogenous (danger signals) stimuli [8]. Yang et al. reported that pyroptosis-related proteins (including caspase-1, caspase-11, and interleukin-1β (IL-1β)) were significantly upregulated in tubular epithelial cells 6 h after renal IRI [9]. Pyroptosis in renal tubular epithelial cells is a critical factor during IRI and CHOP-caspase-11 is induced by excessive activation of the endoplasmic reticulum [9].

Pyrogenesis protects against intracellular infection by eliminating damaged cells and triggering an inflammatory response [10]. Wang et al. reported that cholecalciferol has the potential to preserve renal function in patients with AKI by reducing pyroptosis [10]. However, the specific pathways of pyroptosis during I/R damage are under-researched. A class of small noncoding RNAs known as microRNAs (miRNAs) were found to be critical regulators of cellular processes such as differentiation, proliferation, and apoptosis. Studies had demonstrated that pyroptosis is regulated by miRNAs [11–15]. MiR-214 suppresses proliferation and migration through inhibiting caspase 1-mediated pyroptosis in glioma U87 and T98G cells [16], which opens the avenue for a novel therapy to treat gliomas. As another example, miRNA-124 provides neuroprotection against pyroptosis during cerebral IRI through suppressing STAT3 pathway.

One type of DEAD-box helicase family member, a novel identified NOD-like receptor family pyrin domain containing 3 (NLRP3) inflammasome component, the human DEAD-box Helicase 3 X-Linked (DDX3X) was found to act as a crucial checkpoint for pro-apoptosis in stressed microenvironments. Previous studies had explored the vital role of DDX3X in transcription regulation in the nucleus to translation initiation and stress granule formation [17], and found that DDX3X played a crucial role in innate immunity, as well as tumorigenesis and viral infections [18]. DDX3X modulated the crosstalk between cellular stress responses and innate immune signaling pathways by acting as an essential factor in the activation of the NLRP3 inflammasome. It was found that miRNAs regulated DDX3X levels both directly and indirectly. DDX3X bound to the miR-20a locus and regulated its expression level as an RNA-binding protein [19]. The depletion of DDX3X led to a reduction in miR-20a pri/pre/mature species, indicating that DDX3X took part in pri-miRNA production or stability. DDX3X was also found to reduce DNA methyltransferase 3A binding and hypermethylation of the promoter regions of tumor-suppressive miR-122, miR-200b, and miR-145 [19, 20]. Feng et al. found that DDX3X deficiency attenuated cardiomyocytes pyroptosis by harboring activation of NLRP3 inflammasome [21]. Xu et al. revealed that the downregulation of miRNA-155-5p participated in the process of promoting the pyroptosis of breast cancer cells [22]. Klimczak et al. reported that the plasma microRNA-155-5p was increased in patients with chronic kidney disease and nocturnal hypertension [23]. Despite these advances, the role by which miRNA-155-5p regulates pyroptosis through its downstream targets warrants further investigation. Whether miRNA-155-5p promotes renal tubular cell pyroptosis by interplaying with DDX3X and activating NLRP3 inflammasome remains unknown.

## RESULTS

### Pathological changes

**Figure 1:**
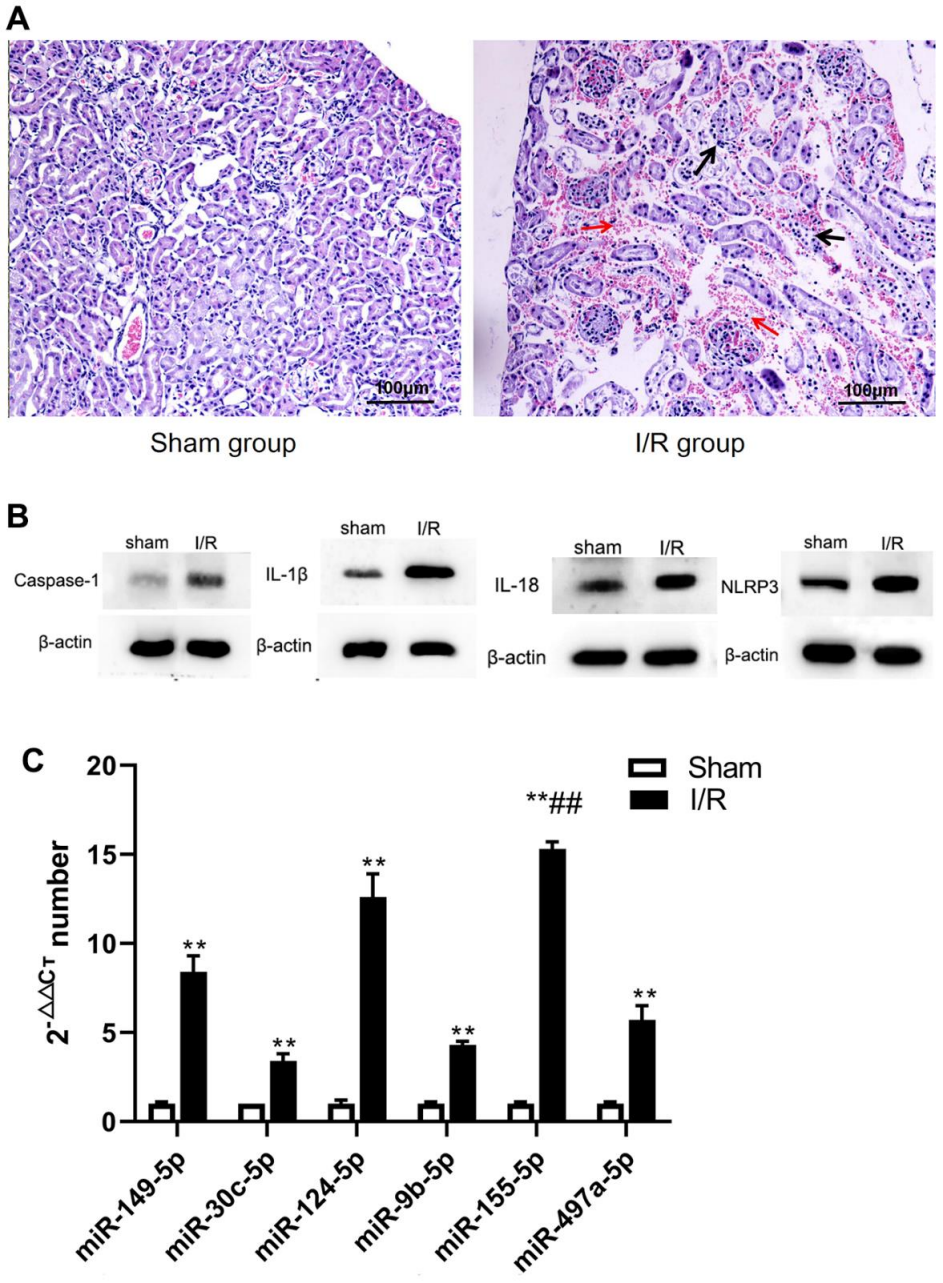
**The changes of renal pathological, microRNA and pyroptosis related factors in sham and I/R group.** (**A**) Pathological changes of renal tissue (400 ×). (**B**) microRNA changes. (**C**) Expression of caspase-1, IL-1β, IL-18. and NLRP3. (**p<0.01, compared with control group).

To investigate the pyroptosis of renal tissue after IRI, a mouse renal IRI model was successfully constructed through the occlusion of the renal pedicles. Then, histological samples were obtained after reperfusion (24 h), and changes in the mouse renal tissue structure were analyzed. In the sham group (S group), the renal tissue structure was intact and clear, the renal tubules were closely arranged, the boundary was clear, and no inflammatory cell infiltration was observed. The tissue was severely damaged after reperfusion in the I/R group. The tissue arrangement was loose with an enlarged tissue gap, and the tissue exhibited hyperemia, swelling, and inflammatory cell infiltration. The results indicated that renal I/R mice were successfully modeled and constructed (Figure 1A).

### Changes in correlation proteins in I/R pyroptosis

To explore pyroptosis of renal tissue after injury, we evaluated relevant proteins, including caspase-1, NLRP3, IL-1β, and IL-18. The levels of these proteins were higher in the I/R group than in the S group. (t=-13.870, -2.833, -7.840, -15.456, respectively; *P*<0.01) (Figure 1B). This data suggested that IRI caused NLRP3-mediated pyroptosis.

### microRNA analysis

The renal expression of miR-149-5p, miR-30c-5p, miR-124-5p, miR-9b-5p, miR-155-5p, and miR-497a-5p, all of which regulated renal pyroptosis, were screened and detected by real-time reverse transcription polymerase chain reaction (RT-PCR). The I/R group was associated with significantly higher expression of miR-149-5p, miR-30c-5p, miR-124-5p, miR-9b-5p, miR-155-5p, and miR-497a-5p (t=-19.805, -15.271, -21.798, -46.087, -77.322, -12.236, respectively; *P*<0.01). The expression of miR-155-5p in the I/R group was higher than that in S group (Figure 1B). In subsequent experiments, miR-155-5p was explored to determine whether it regulated the progression of pyroptosis in renal IRI (Figure 1C).

### miR-155 acted with DDX3X

**Figure 2:**
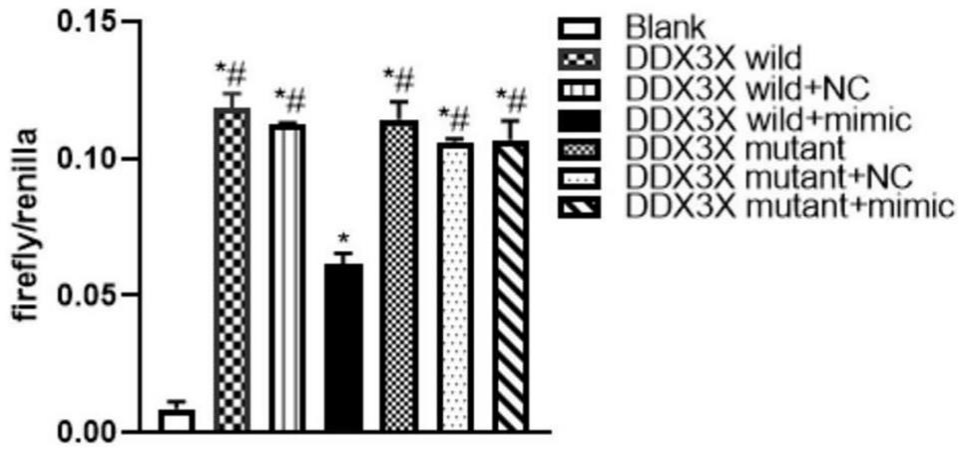
**Luciferase assay of miR-155 directly targeted DDX3X.** (*p<0.05, compared with Blank group; #*p*<0.05, compared with DDX3X wild+mimic group).

Online analysis (http://starbase.sysu.edu.cn/) revealed a specific binding region on the 3′UTR of DDX3X, which was the same with the miR-155-5p sequence. To test whether DDX3X acted as a direct target interplaying with miR-155-5p, a firefly luciferase reporter containing the DDX3X sequence that was the potential interplaying site was constructed. The data showed that the luciferase activities in all DDX3X wild-type and mutant groups were higher (F=246.441, *P*<0.05) than in the negative control (NC) group. Compared with the luciferase activity of the DDX3X wild+mimic group, the activities of wild-type DDX3X and mutant groups were higher (F=59.586, *P*<0.05). The results showed that DDX3X was inhibited by miR-155-5p mimic (Figure 2). We hypothesized that the interplaying effect between DDX3X and miR-155-5p could regulated renal pyroptosis during IRI.

### miR-155-5p regulated HK2 cell pyroptosis via DDX3X

**Figure 3:**
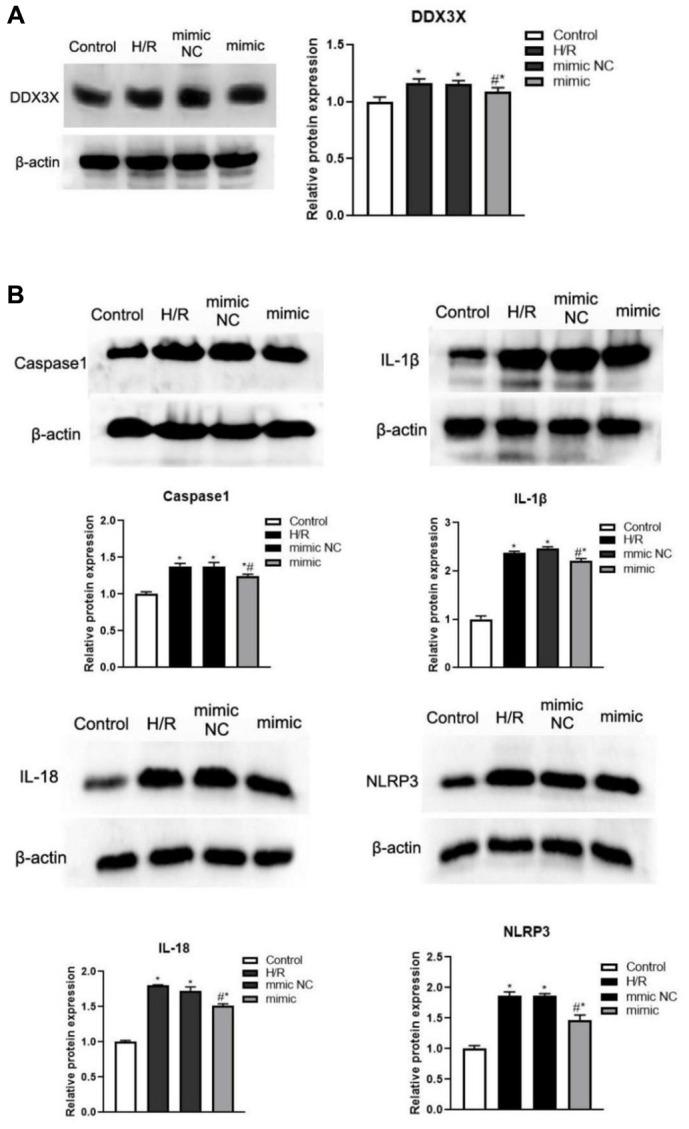
**Changes of DDX3X and pyroptosis related factors in all HK2 groups.** (**A**) Expression of DDX3X. (**B**) Expression of NLRP3, caspase-1, IL-1β, and IL-18 (*p<0.05, compared with the Blank group, #*p*<0.05, Compared with the H/R group and the miR-155-5p mimic NC group).

**Figure 4:**
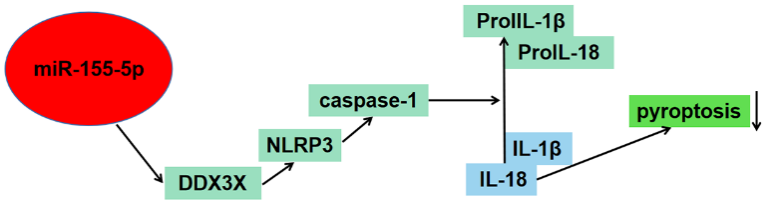
Schematic figure illustrated the potential role of miR-155-5p in regulating pyroptosis of renal cells in I/R injury via the DDX3X/NLRP3/caspase-1 pathway.

To determine whether miR-155-5p combine with DDX3X, inhibited pyroptosis, and alleviated IRI, we overexpressed miR-155-5p mimic in all HK-2 cell groups. Then, we detected the expression of DDX3X and pyroptosis-related factors. Compared with the expression in the control group, the expression of DDX3X, caspase-1, NLRP3, IL-1β, and IL-18 in all hypoxia-reoxygenation (H/R) groups were more upregulated (F=15.189, 56.900, 670.126, 337.487, 163.0023, respectively; *P*<0.05) (Figure 3A). Moreover, the levels of DDX3X, caspase-1, NLRP3, IL-1β, and IL-18 were significantly lower in the miR-155-5p group than in the H/R and miR-155-5p group. (F=5.448, 9.763, 39.136, 47.818, 49.423, respectively; *P*<0.05) (Figure 3B). These results showed that miR-155-5p inhibited the activation of DDX3X and the expression of NLRP3 and caspase-1 (Figure 4).

**Figure 5:**
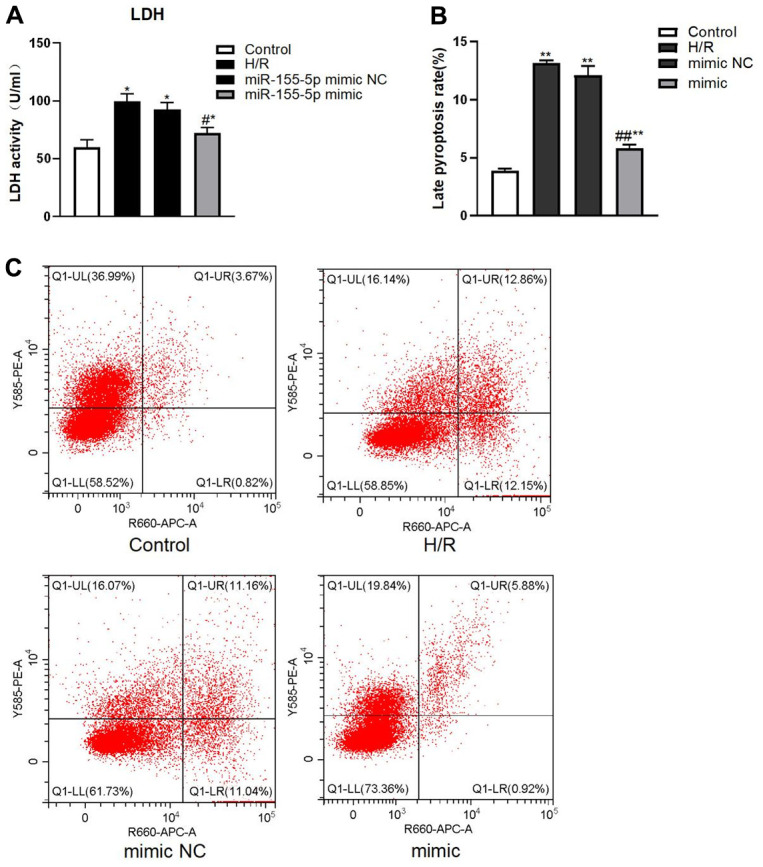
**Changes of LDH and pyroptosis rate in all HK2 groups.** (**A**) Expression of LDH activity. (**B**) Late pyroptosis rate of HK2 cell, (**C**) Flow cytometric graphs showing cell pyroptosis. The red dots in higher right quadrant of each flow cytometry diagram indicate pyroptosis cells. (*p<0.05, **p<0.01, compared with the blank group, #*p*<0.05, ##*p*<0.05, compared with the H/R group and the miR-155-5p mimic NC group).

LDH activity (by enzyme-linked immunosorbent assay (ELISA)) and pyroptosis rate (by flow cytometry) in all groups with H/R were higher than those in the control group (F=49.215 and 305.226, respectively; *P*<0.01) (Figure 5A). LDH activity and pyroptosis rate were significantly lower in the miR-155-5p mimic group than in the H/R group and miR-155-5p mimic NC group (F=31.277 and 179.364, respectively; *P*<0.05) (Figure 5B).

## DISCUSSION

The effect of IRI on renal cells in the proximal tubule is characterized by the damaged brush border, loss of cellular polarity, cellular atrophy, disruption of cell-cell adhesion, and death [24]. Both apoptosis and necrosis are key processes of cell death in renal IRI [25, 26]. Pyroptosis is a compelling and critical mechanism of cell death after IRI, which is characterized by the continuous expansion of cells until the cell membrane ruptures, leading to the release of intracellular pathogens and pro-inflammatory mediators that activate a strong inflammatory cascade [9, 10, 27–29]. We demonstrated that the IRI was associated with severe damage in the renal tissue, hyperemia, swelling, and inflammatory cell infiltration. Moreover, the expression of NLRP3, caspase-1, IL-1β, and IL-18 in the kidney were all upregulated in the I/R and H/R groups. These results were consistent with those as other IRI inducing pyroptosis of the kidney [9, 10, 27, 28].

More than 10 miRNAs, which serve as noncoding RNAs, can regulate pyroptosis. Few microRNAs can promote pyroptosis progression in certain pathological processes by regulating NLRC4 to facilitate pyroptosis in diabetic nephropathy [30]. As reported in previous studies, miR-1656 upregulated the expression of oxygen-containing reactive species through binding to GPX4, activating the NLRP3 inflammation, and releasing IL-18 and IL-1β to trigger pyroptosis in the nephridial tissue of Se-deficient broilers [31]. Other miRNAs repress pyroptosis progression in some diseases. Through inhibition of CTSB/NLRP3, miR-140-5p suppressed pyroptosis of chondrocyte and alleviated osteoarthritic inflammation [32], and miR-223-3p reduced pyroptosis in fibroblast-like synoviocytes through targeting NLRP3 [33]. Thus, the role of miR-155-5p played in renal I/R inflammation-induced pyroptosis had not been well understood. In this study, it was found that the expression of miR-155-5p was higher after the renal I/R than that of other microRNAs; thereby indicating that miR-155-5p might play important role in renal I/R damage.

Through bioinformatics analysis, we confirmed that miR-155-5p combined with the 3′UTR region of DDX3X. DDX3X took part in various aspects of eukaryotic RNA metabolism [34, 35]. DDX3X was critical for the activation of NLRP3 inflammasome pyroptosis [17]. It was required for NLRP3 inflammasome activation by both potassium efflux-dependent and independent triggers [17]. Swanson et al. reported that NLRP3 recognized endogenous and exogenous danger signals, activating of NLRP3 inflammasome [36]. NLRP3 recognized stimuli such as pathogens, metabolites, toxins, nucleic acids, and ATP. NLRP3 inflammasome assembly induced upregulation of the pro-inflammatory cytokines IL-18 and IL-1β and gasdermin D-mediated pyroptosis. Therefore, the mechanisms by which miR-155-5p regulates pyroptosis through its downstream targets warranted further investigation.

Overall, our findings from the cell luciferase assay confirmed that miR-155-5p downregulated the level of DDX3X. Notably, NLRP3-related pyroptosis was downregulated. When HK-2 cells were reoxygenated after hypoxia, the expression of DDX3X, NLRP3, caspase-1, IL-1β, and IL-18 in all H/R groups were more significant than those in the control group. This indicates that the DDX3X/NLRP3 pathway might be involved in the pyroptosis of HK cells. Whereas, miR-155-5p overexpression decreased the level of DDX3X, NLRP3, caspase-1, IL-1β, and IL-18, indicating that miR-155-5p alleviated H/R-induced apoptosis. It was reported that miR-155, via direct targeting of Foxo3a, promoted renal apoptosis, suggesting that miR-155-5p played a dual role in pyroptosis caused by renal I/R [37]. At the same time, in this paper, miR-155-5p down-regulated DDX3X and inhibits the occurrence of pyroptosis, which was a unique phenomenon. This showed the complexity of microRNA regulation in pyroptosis. We are not sure which any microRNA must have positive or negative feedback effect.

In conclusion, current evidence had revealed that pyroptosis of renal cells in IRI was regulated by miR-155-5p via direct targeting of the DDX3X/NLRP3/caspase-1 pathway. Our findings confirmed that miR-155-5p downregulated the level of DDX3X to inhibit pyroptosis. Thus, the findings of our study highlighted the potential of mitigating pyroptosis by enhancement of miR-155-5p, which might serve as a promising novel target.

## MATERIALS AND METHODS

### Experimental animals

Fourteen healthy 7-week-old male C57BL/6 mice were purchased from the Medical Experimental Animal Center of Lanzhou University, China. Mice were divided into sham operation (S group, n=7) and renal I/R groups (group I/R, n=7). All mouse experiments were implemented in accordance with the standards approved by the Lanzhou University Animal Care and Use Committee.

### The animal model of renal I/R

Intraperitoneal injections of pentobarbital sodium (70mg/kg) was used for anaesthetizing the mice. The mice in the S group underwent surgical procedure through suspending the renal blood supply without occlusion of the renal pedicles. C57BL/6 mice in the I/R group were subjected to renal IRI, which was sustained 20 min followed by reperfusion under body temperature (37° C). The mice from each group (n=7) were sacrificed 24 h after reperfusion to obtain blood and kidney samples.

### *In vitro* H/R injury model

Human renal proximal tubular epithelial cells (HK-2 cells) were treated in DMEM with 10% fetal bovine serum at 5% CO_2_, 95% air atmosphere, and 37° C temperature. A hypoxic reoxygenation (H/R) model was established using mineral oil coverage. HK-2 cells suspension were used to complete culture medium according to 5 × 10^5^ cells/ml, inoculated 2ml of each well into 6-well cell culture plate, when the fusion degree was about 80% at 37° C with 5% CO2, washed the cells twice with PBS, added mineral oil to each well until the cells were completely covered, cultured for 1h at 37° C with 5% CO2, then discarded the mineral oil, and an appropriate amount of PBS was added for rinsing three times; 2ml of serum free DMEM/F12 medium was infused into each well and cultured at 37° C with 5% CO2. To determine the effect of miR-155-5p, a miR-155-5p mimic was transfected into cells (Zhonghong Boyuan Biological Technology, Jiangxi, China) 12 hours before H/R treatment. Four groups were used to analyze the differences: control group (no H/R and miR-155-5p mimic transfected), H/R group (H/R), mimic NC group (H/R and miR-155-5p mimic control transfected), and mimic group (H/R and miR-155-5p mimic transfected).

### Histopathology

Renal tissue samples were fixed in 10% formalin for 24 h and embedded in paraffin. The sections were stained with hematoxylin and eosin using standard histological techniques. The samples were analyzed under a light microscope.

### Western blotting analysis

Tissues and cells were lysed in ice-cold RIPA lysis buffer (Solarbio, Beijing, China). Samples (30 μg per lane) were separated by 12% SDS-PAGE and transferred to polyvinylidene difluoride (PVDF) membranes (Immobilon-P; Millipore, Bedford, MA, USA). After the 1 h blocking with 5 % nonfat milk, membranes were probed with the following primary antibodies at 4° C overnight: rabbit anti-caspase 1 (1:1500; Santa Cruz Biotechnology Inc., Dallas, TX, USA), anti-NLRP3 (1:1000; Biosynthesis Biotechnology, Beijing, China), anti-DDX3X (1:1000; Biosynthesis Biotechnology, Beijing, China), anti-IL-1β (1:1000; Cell Signaling Technology, Danvers, MA, USA), anti-IL-18, and mouse anti-β-actin (1:3000; BOSTER, Wuhan, China). After five washes with PBS-Tween 20, horseradish peroxidase (HRP)-conjugated goat antibodies (1:3000, Abcam, Cambridge, MA, USA) were added. The membranes were then incubated with horseradish peroxidase-conjugated goat anti-rabbit IgG (1:3000; Biosynthesis Biotechnology, Beijing, China). These results were visualized and then quantified using Quantity One software (Bio-Rad, Hercules, CA, USA) by scanning the exposed X-ray films.

### Quantitative real-time polymerase chain reaction

RNA was extracted from frozen kidney tissues according to the instructions of the miRNeasy Mini Kit. MiR-149-5p, miR-30c-5p, miR-124-5p, miR-9b-5p, miR-497a-5p, and miR-155-5p in the mouse kidney tissues were measured through reverse transcription and amplification (Table 1).

**Table 1:** Primers of microRNAs.

**Gene name**	**Forward primer (5’-3’)**	**Reverse primer (5’-3’)**
miR-149-5p	CGTCTGGCTCCGTGTCTTC	GTCGTATCCAGTGCAGGGTCCGAGGTATTCGCACTGGATACGACGGGAGT
miR-30c-5p	GCGCGTGTAAACATCCTACACT	GTCGTATCCAGTGCAGGGTCCGAGGTATTCGCACTGGATACGACGCTGAG
miR-124-5p	CGCGTGTTCACAGCGGAC	GTCGTATCCAGTGCAGGGTCCGAGGTATTCGCACTGGATACGACATCAAG
miR-9b-5p	GCGCGTTCGGTTATCTAGCT	GTCGTATCCAGTGCAGGGTCCGAGGTATTCGCACTGGATACGACTCATAA
miR-497a-5p	CGCAGCAGCACACTGTGG	GTCGTATCCAGTGCAGGGTCCGAGGTATTCGCACTGGATACGACTACAAA
miR-155-5p	GCGCGTTAATGCTAATTGTGAT	GTCGTATCCAGTGCAGGGTCCGAGGTATTCGCACTGGATACGACACCCCT

### Luciferase test

The 3’-UTR of DDX3X and miR-155-5p binding sites were fused downstream of the luciferase reporter in the pmirGLO dual-luciferase miRNA Target Expression Vector (Zhonghong Boyuan Biological Technology, Jiangxi, China). A dual-luciferase reporter assay was performed in 293T cells. The cells were divided into a black group (no transfer), DDX3X wild group (DDX3X wild dual-luciferase vector transferred), DDX3X wild +NC group (DDX3X wild dual-luciferase vector, and miR-155-5p mimic control transferred), DDX3X wild+mimic group (*DDX3X* wild dual-luciferase vector and miR-155-5p mimic transferred), DDX3X mutant group (*DDX3X* mutant dual-luciferase vector transferred), DDX3X mutant+NC group (*DDX3X* mutant dual-luciferase vector transferred and miR-155-5p mimic control transferred), and DDX3X mutant+mimic group (*DDX3X* mutant dual-luciferase vector and miR-155-5p mimic transferred).

### Lactate dehydrogenase (LDH) activity assay

HK-2 cells of each group were prepared into 1×10^4^/mL cell suspension with the extraction liquid. Cells were lysed by ultrasonication at 4° C, centrifuged at 8000 *g* for 10 min. The supernatant was separated and calculated for the determination of LDH activity through the LDH kit (ab102526, Abcam, U.K.). The optical density was measured at 440 nm using a microplate reader, and LDH activity was calculated by spectrophotometrically following the manufacturer’s instructions.

### Flow cytometry

Cell pyroptosis was detected by flow cytometry using the Annexin V-PE/7AAD kit (Solarbio Science and Technology Co., Beijing, China) according to the manufacturer’s protocol. Briefly, HK-2 cells were seeded at 1×10^6^/mL and plated on a 96-well culture plate. After treatment with drugs for 48 h, they were harvested and washed twice with the binding buffer (10 mM Na-HEPES, 140 mM NaCl, 2.5 mM CaCl2, pH 7.4). Cell pellets obtained by centrifugation (200g for 10 min) and 1 mL of cell suspension was added to the cleaved capase-1 reagent before incubation. Then, 10 μL PI was added, and the pyroptosis rate was detected by the FACSVerse flow cytometer (BD Biosciences, San Jose, CA, USA). Data acquisition and analysis were performed using the Flowjo software (BD Biosciences, San Jose, CA, USA). Each group had three parallel controls, and the process was duplicated three times.

### Statistical analysis

All experiments were repeated in triplicate. Data were reported as means ± standard deviation (SD). The independent samples t-test and one-way analysis of variance (ANOVA) were applied to compare the data, respectively. and determine the statistical significance between groups. SPSS (version 19.0 for Windows, SPSS Inc., Chicago, IL, USA) was used for analysis. And *P<* 0.05 represented statistically significant.

### Data availability

The original contributions presented in the study were directed to the corresponding authors.
